# Orexin antagonists for neuropsychiatric disease: progress and potential pitfalls

**DOI:** 10.3389/fnins.2014.00036

**Published:** 2014-02-25

**Authors:** Jiann Wei Yeoh, Erin J. Campbell, Morgan H. James, Brett A. Graham, Christopher V. Dayas

**Affiliations:** Neurobiology of Addiction Laboratory, The Centre for Translational Neuroscience and Mental Health Research, School of Biomedical Sciences and Pharmacy, University of Newcastle and the Hunter Medical Research InstituteNewcastle, NSW, Australia

**Keywords:** hypothalamus, orexin, stress, anxiety, depression, cocaine, reinstatement, reward seeking

## Abstract

The tight regulation of sleep/wake states is critical for mental and physiological wellbeing. For example, dysregulation of sleep/wake systems predisposes individuals to metabolic disorders such as obesity and psychiatric problems, including depression. Contributing to this understanding, the last decade has seen significant advances in our appreciation of the complex interactions between brain systems that control the transition between sleep and wake states. Pivotal to our increased understanding of this pathway was the description of a group of neurons in the lateral hypothalamus (LH) that express the neuropeptides orexin A and B (hypocretin, Hcrt-1 and Hcrt-2). Orexin neurons were quickly placed at center stage with the demonstration that loss of normal orexin function is associated with the development of narcolepsy—a condition in which sufferers fail to maintain normal levels of daytime wakefulness. Since these initial seminal findings, much progress has been made in our understanding of the physiology and function of the orexin system. For example, the orexin system has been identified as a key modulator of autonomic and neuroendocrine function, arousal, reward and attention. Notably, studies in animals suggest that dysregulation of orexin function is associated with neuropsychiatric states such as addiction and mood disorders including depression and anxiety. This review discusses the progress associated with therapeutic attempts to restore orexin system function and treat neuropsychiatric conditions such as addiction, depression and anxiety. We also highlight potential pitfalls and challenges associated with targeting this system to treat these neuropsychiatric states.

## Overview—the orexin system in brief

First described in 1996, the orexins are two neuropeptides expressed by a few thousand neurons within the perifornical area (PFA), the dorsomedial hypothalamus (DMH) and the lateral hypothalamus (LH) (de Lecea et al., 1998; Sakurai et al., 1998). The binding target of these ligands, termed orexin A and B, are two G-protein coupled receptors OxR1 and OxR2 (de Lecea et al., 1998; Sakurai et al., 1998). Orexin A is non-selective for both OxR1 and OxR2 whereas orexin B is more selective for OxR2 (Sakurai et al., 1998; Ammoun et al., 2003). A key feature of this relatively small population of neurons is their widespread projections throughout the brain, including other hypothalamic nuclei, the midline paraventricular thalamus (PVT), brain stem nuclei and a number of structures involved in reward behavior including the ventral tegmental area (VTA) and nucleus accumbens shell (NACs) (Peyron et al., 1998). In these projection areas, the expression of orexin receptor subtypes is partially overlapping, however, some regions preferentially express one receptor subtype, presumably providing some degree of selectivity (certainly in terms of potential pharmacological selectivity of different target regions). For example, the prefrontal cortex predominantly expresses OxR1, whereas the nucleus accumbens (NAC) mainly expresses OxR2 (Marcus et al., 2001).

Consistent with the widespread projections of these neurons, orexins have been implicated in a number of physiological functions, including regulation of sleep (Chemelli et al., 1999), energy metabolism (Burdakov et al., 2005), arousal (Sutcliffe and de Lecea, 2002; Taheri et al., 2002), behavioral and neuroendocrine responses to stress (Ida et al., 2000; Furlong et al., 2009) and reward-seeking behavior (Boutrel et al., 2005; Harris et al., 2005; Lawrence et al., 2006; Marchant et al., 2012). The role of orexin in this diverse range of functions has been reviewed extensively elsewhere (Boutrel and de Lecea, 2008; Aston-Jones et al., 2010; Boutrel et al., 2010; Lawrence, 2010; James et al., 2012; Mahler et al., 2012). In this review, we will highlight new research focused on changes in the intra-hypothalamic LH-orexin circuitry induced by drugs of abuse and stress. We will also outline recent data highlighting the clinical potential of single and dual orexin receptor antagonists (SORAs and DORAs) for neuropsychiatric conditions including addiction, anxiety and depression. However, we also discuss recent findings indicating that several challenges must be overcome for the therapeutic value of SORAs and DORAs to be fully realized.

## Cellular and molecular evidence for targeting the LH-orexin system in drug addiction

Until recently, work on the relationship between the orexin system and addiction has been heavily focused on the antagonism of orexin actions in downstream projection areas (James et al., 2011; Brown et al., 2013; Mahler et al., 2013). At the same time, the issue of how drugs of addiction might alter the properties of orexin neurons has been largely overlooked. Interestingly, gene expression analysis has shown that the LH is highly transcriptionally responsive to addictive drugs (Ahmed et al., 2005). Ahmed et al. (2005) found that rats given extended access to cocaine displayed a profound increase in both pre- and post-synaptic markers of plasticity in the LH. Further, studies from the feeding literature suggest that excitatory inputs onto orexin neurons can undergo significant rewiring in response to food-deprivation and re-feeding. These results suggest that the LH orexin circuitry is likely to undergo experience-dependent neuroplasticity in response to cocaine treatment.

Given the above evidence, our group has undertaken a series of studies to assess how drugs might remodel LH orexin circuits. In these experiments, rats were exposed to 7 days of cocaine injections or allowed to self-administer cocaine for 14 days before excitatory synaptic transmission was assessed in LH slices. This work showed that both experimenter- and self-administered cocaine significantly increased excitatory drive in the LH through pre-synaptic mechanisms, assessed using mEPSCs frequency, amplitude and paired pulse ratio (Yeoh et al., 2012). Consistent with our electrophysiological findings, the number of putative excitatory but not inhibitory inputs onto PFA/LH cells were significantly increased, as measured by immunolabeling for vesicular glutamate transporter 2 (VGLUT2) or vesicular GABA transporter (VGAT; Yeoh et al., 2012). Importantly, a population of recorded neurons that were recovered with neurobiotin labeling and immunolabeled for orexin confirmed that these increases in excitatory drive occurred in orexin neurons.

**Figure 1:**
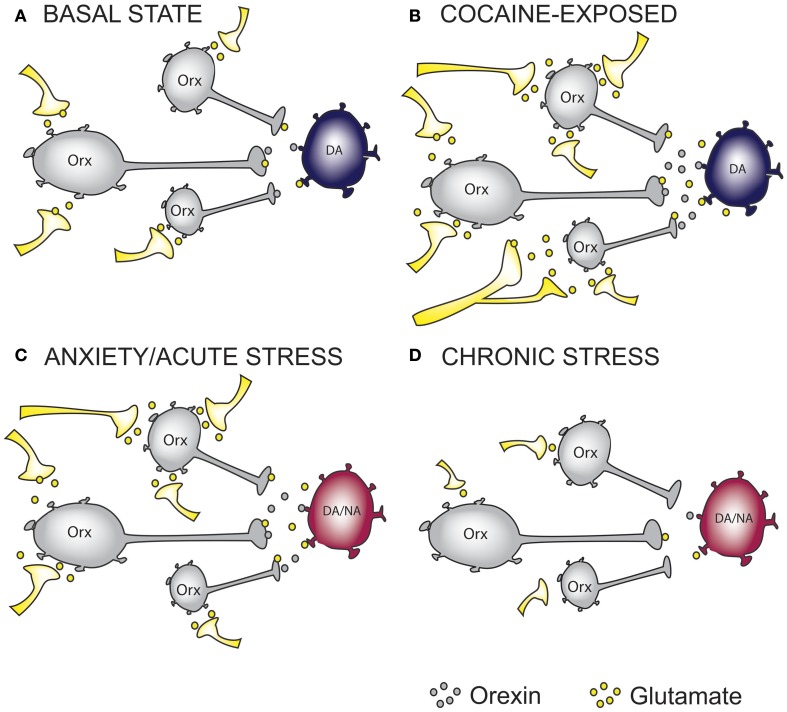
**The effect of cocaine and stress exposure on orexin neurons. (A)** Under basal conditions, orexin (Orx) neurons in the lateral hypothalamus receive excitatory inputs, thus providing “normal” glutamatergic and orexinergic (excitatory) tone onto downstream projection targets such as ventral tegmental area (VTA) dopamine (DA) neurons. **(B)** Based on recent studies, we propose that chronic cocaine-exposure rewires glutamatergic inputs onto Orx neurons resulting in increased excitatory drive onto DA neurons in the VTA. **(C)** Anxiety and acute stress are able to cause enhanced excitatory drive to DA neurons in the VTA and Noradrenergic (NA) neurons in the Locus Coeruleus (LC), an important projection target of orexin neurons. **(D)** Glutamatergic inputs onto Orx neurons in chronically stressed animals are impaired resulting in reduced excitatory drive onto DA and/or NA neurons. On the basis of this simplified hypothetical mechanistic rationale, orexin receptor antagonism represents a possible therapeutic intervention to treat anxiety and addictions. In the case of reduced orexin system activity, as may occur in some forms of depression or in response to chronic stress, mechanisms to augment orexin function may be required. Whether chronic modulation of these systems can be achieved without precipitating the expression of an alternate neuropsychiatric state warrants careful consideration.

Somewhat surprisingly, a recent study by a different group failed to find evidence of pre-synaptic plasticity in orexin neurons after 3 days of cocaine exposure (Rao et al., 2013). However, this regimen of cocaine exposure did promote long-term potentiation (LTP) at glutamate synapses onto orexin neurons, which persisted for more than 5 days post-withdrawal (Rao et al., 2013). The differences in the Rao et al. (2013) study and our own may in part be explained by differences in experimental procedures. In our study, we carried out both intraperitoneal (i.p) injections and cocaine self-administration in rats as compared to the Rao study, which employed a conditioned place preference (CPP) model whereby mice received only experimenter-administered cocaine injections. Animals that underwent i.p injections in our study received a slightly higher dose of cocaine for a longer period of time as compared to Rao and colleagues (15 mg/kg/7 days vs. 10 mg/kg/3 days). Further, our animals which were trained to self-administer cocaine (14 days, 0.25 mg/0.1 ml/infusion) did not show signs of post-synaptic adaptations. Therefore, species differences are unlikely to have contributed to this disparity. Furthermore, preliminary experiments in our laboratory indicate similar pre-synaptic effects in mice. A more likely explanation might be that in our study, all experimental procedures were carried out during the active (dark) phase while CPP procedures performed by Rao et al. (2013) occurred during the inactive (light) phase. Despite these differences in experimental procedures, both experiments indicate that cocaine induces synaptic plasticity in LH orexin circuitry (Figures 1A, B). These data, which indicate that addiction leads to an overstimulation of orexin neurons, may provide a mechanistic rationale for antagonizing the downstream actions of enhanced orexin signaling in addiction using SORAs and DORAs.

Furthermore, evidence exists that altered feeding behavior modifies LH orexin neuron circuitry at a cellular level. Specifically, work using an orexin-GFP mouse to selectively study the properties of orexin neurons showed an increase in miniature excitatory post-synaptic currents (mEPSCS), a measure of increased synaptic drive, in LH slices of mice that underwent mild food restriction for 24 h, as compared to normally-fed controls. Consistent with these electrophysiological findings, and similar to our LH addiction experiments, overnight food restriction promoted the formation of excitatory VGLUT2 synapses onto orexin neurons. These cellular effects of food restriction were rapidly reversed by re-feeding (Horvath and Gao, 2005), contrasting the addiction work where comparable cellular perturbations persisted beyond the removal of drug. Together, these behavioral and cellular studies highlight the potential impact for SORAs and DORAs on appetite and natural rewards.

Importantly, the source of the enhanced excitatory drive to orexin neurons remains to be determined. The LH receives significant glutamatergic inputs from other brain regions such as the prefrontal cortex, lateral septum and basolateral amygdala. For example, Morshedi and Meredith (2008) have shown that a sensitizing regimen of amphetamine upregulates Fos immunoreactivity in medial prefrontal cortex neurons projecting to the LH. Further, cocaine CPP has also been demonstrated to produce increased Fos immunoreactivity in the lateral septum, another area that projects to the LH (Sartor and Aston-Jones, 2012). It is also worth noting the possibility that local glutamatergic neurons may provide positive feedback to the orexin system in cocaine-exposed animals (Li et al., 2002; Jennings et al., 2013). Nonetheless, further studies are needed to confirm the likely source of these increased glutamatergic inputs onto orexin neurons in response to cocaine exposure and to determine how other classes of drugs might also rewire LH-orexin circuits. A final point that should be noted is that recent elegant work by Burdakov's group has demonstrated the existence of two subpopulations of orexin neurons in the LH (termed H-type and D-type), which can be distinguished electrophysiologically and anatomically (Schöne et al., 2011). It will be important for future studies to determine if drugs of abuse (or stress) differentially affect these orexin cell subtypes.

## Effects of SORAs and DORAs on drug-seeking behaviors

A number of SORAs now exist, the most common being those that target the OxR1. These include SB-334867 (Porter et al., 2001; Smart et al., 2001), SB-674042 (Langmead et al., 2004), SB-408124 (Langmead et al., 2004), SB-410220 (Porter et al., 2001; Langmead et al., 2004), GSK1059865 (Gozzi et al., 2011) and, most recently, ACT-335827 (Steiner et al., 2013a) all of which have at least 50-fold selectivity for OxR1 over OxR2 (Scammell and Winrow, 2011; Zhou et al., 2011). Amongst these compounds, SB-334867 has been most widely studied in terms of behavioral pharmacology, largely due to the high selectivity, potency and availability of this drug (Scammell and Winrow, 2011; Zhou et al., 2011).

A large number of studies have demonstrated that OxR1 antagonists are effective at blocking addiction-related behaviors across a range of drug classes. With respect to self-administration behavior, treatment with SB-334867 effectively attenuates ethanol and nicotine self-administration under both low-effort fixed ratio (FR) and higher-effort progressive ratio (PR) schedules (Lawrence et al., 2006; Schneider et al., 2007; Hollander et al., 2008; LeSage et al., 2010; Jupp et al., 2011; Martin-Fardon and Weiss, 2012). Interestingly, in the case of cocaine, SB-334867 has no effect on self-administration behavior under FR1 or FR3 conditions (Smith et al., 2009; Espana et al., 2010) but does attenuate self-administration under higher-effort schedules, including FR5 (Hollander et al., 2012) and PR (Borgland et al., 2009; Espana et al., 2010). These findings imply that in general, the orexin system has limited actions on the primary rewarding effects of cocaine, but is important to overcome increased motivational demands or effort to procure rewards. In contrast, SB-334867 effectively blocks the expression of CPP for amphetamine (Hutcheson et al., 2011) and morphine (Sharf et al., 2010), a widely used measure of the rewarding effects of drugs of abuse. Systemic SB-334867 treatment also attenuates reinstatement of cocaine-seeking behavior elicited by drug cues (Smith et al., 2009), contexts (Smith et al., 2010) and footshock stress (Boutrel et al., 2005), but not a cocaine prime (Mahler et al., 2013). Similarly, SB-334867 attenuates cue- and stress-induced reinstatement of alcohol seeking (Lawrence et al., 2006; Richards et al., 2008; Jupp et al., 2011) as well as cue-induced, but not primed, heroin seeking (Smith and Aston-Jones, 2012). SB-334867 also attenuates cue- (Plaza-Zabala et al., 2013), but not footshock- (Plaza-Zabala et al., 2010) induced reinstatement of nicotine seeking—the latter result being a particularly surprising outcome. Thus, a wealth of preclinical studies support the premise that the use of OxR1 SORAs have the potential to decrease addiction-related behaviors, suggesting they may have clinical value in preventing relapse in abstinent patients.

In contrast to OxR1 antagonists, fewer studies have assessed the effects of selective OxR2 antagonists on addiction-related behaviors. This is likely due to the more recent development of these compounds and a predicted increase in the likelihood of sedation (Zhou et al., 2011). Selective OxR2 antagonists include JNJ-10397049 (McAtee et al., 2004; Dugovic et al., 2009), EMPA (Malherbe et al., 2009a), and TCS-OX2-29 (Hirose et al., 2003), all of which have at least a 250-fold selectivity for OxR2 (Scammell and Winrow, 2011; Zhou et al., 2011). Studies exploring the effects of these OxR2 SORAs have reported less consistent effects on drug-motivated behaviors compared to the OxR1 SORA SB-334867. For example, Shoblock et al. (2010) showed that JNJ-10397049 dose-dependently deceased ethanol self-administration, as well as the acquisition, expression and reinstatement of ethanol CPP. In contrast, Brown et al. (2013) showed that TCS-OX2-29 attenuated ethanol self-administration but had no effect on cue-induced reinstatement of extinguished ethanol seeking. Interestingly, these authors identified the nucleus accumbens core (NACc) as an important site for OxR2 signaling, as infusions of TCS-OX2-29 into the NACc, but not shell, reduced ethanol self-administration. In contrast, OxR2 SORAs have been shown to have no effect on cocaine self-administration or reinstatement (Smith et al., 2009) or the expression of nicotine withdrawal symptoms (Plaza-Zabala et al., 2012). Thus, regarding SORAs, the case for OxR1-based therapies in treating addiction is better developed than for antagonists targeting OxR2.

With respect to DORAs, a large number of these compounds have been developed, prompted largely by their potential as a novel treatment for insomnia. Indeed, at least three pharmaceutical companies (GSK, MERCK, Actelion) have initiated clinical trials investigating the utility of DORAs in modulating the sleep-wake cycle. These DORAs include almorexant (ACT-078573; Brisbare-Roch et al., 2007; Malherbe et al., 2009b); suvorexant (MK-4305; Cox et al., 2010), Merck DORA-1 (Bergman et al., 2008), Merck DORA-5 (Whitman et al., 2009), and SB-649868 (Renzulli et al., 2011). Only a limited number of studies have examined the effects of DORAs on addiction-related behaviors, with this work focusing almost exclusively on almorexant. For example, systemic and intra-VTA almorexant treatment was shown to attenuate ethanol self-administration (Srinivasan et al., 2012). Similarly, systemic almorexant blocked nicotine self-administration behavior (LeSage et al., 2010). Interestingly, whilst almorexant attenuated the expression of CPP to high doses of cocaine and amphetamine, it had no effect on morphine CPP (Steiner et al., 2013c). This study also showed that almorexant reduced the expression of behavioral sensitization to morphine but not to cocaine or amphetamine (Steiner et al., 2013c).

## Effect of SORAs and DORAs on food and natural reward-seeking behavior

An important consideration with respect to the potential clinical application of orexin receptor antagonists is the effect of these compounds on other appetitive behaviors, including food seeking. Indeed, studies carried out immediately following the discovery of the orexin peptides firmly implicated orexin in feeding behavior, with intracerebroventricular (i.c.v.) infusions of orexin shown to increase food consumption (Sakurai et al., 1998) whereas systemic treatment with SB-334867 was found to block feeding behavior (Haynes et al., 2000).

Since these initial demonstrations, subsequent studies have sought to investigate whether doses of orexin receptor antagonists that are required to block drug seeking also affect feeding behavior. For example, Martin-Fardon and colleagues recently showed that systemic administration of SB-334867 (1–10 mg/kg, i.p) attenuated reinstatement of cocaine seeking, but not sweetened-condensed milk seeking, elicited by discriminative cues (Martin-Fardon and Weiss, 2014). Similarly, Jupp et al. (2011) showed that systemic injections of SB-334867 (5 mg/kg) were sufficient to reduce responding for both ethanol and sucrose under an FR3 schedule of reinforcement. However, SB-334867 (5 mg/kg) attenuated responding for ethanol but not sucrose under a PR schedule. The authors suggested that the contribution of orexin A to motivation for alcohol is independent of non-specific effects on appetitive drive. Likewise, Hollander et al. (2012) showed that systemic injections of SB-334867 (2–4 mg/kg) reduced cocaine self-administration, but had no effect on responding for food rewards under an FR5 schedule. Comparable effects have also been shown with doses of SB-334867 that attenuate responding for nicotine (LeSage et al., 2010). Further, we have previously reported that intra-VTA infusions of SB-334867, at doses that suppress cue-induced cocaine seeking, had no effect on reinstatement for a natural reward (sweetened condensed milk; James et al., 2012). With respect to OxR2 antagonists, Brown et al. (2013) showed that central infusions of 100 μ g TCS-OX2-29 reduced self-administration of ethanol, but had no effect on sucrose self-administration. In contrast however, LeSage et al. (2010) showed that systemic treatment with the DORA almorexant attenuated responding for both food pellets and nicotine. Similarly, systemic almorexant suppressed ethanol self-administration, and responding for sucrose (Srinivasan et al., 2012).

Taken together, these data indicate that a putative therapeutic window exists in which SORAs could be used to treat addiction-relevant behaviors, including ‘relapse,’ without producing ‘off-target’ effects on natural reward-seeking behavior. Conversely, it appears likely that the use of DORAs in the treatment of addiction may be associated with a risk of interfering with natural appetitive processes or promoting sedation. It is also important to acknowledge that there are very few studies that have assessed the effect of subchronic or chronic orexin receptor antagonism on drug or food-motivated behavior (discussed below).

## Recent progress implicating the orexin system in stress-related neuroendocrine responses

Activation of the hypothalamic-pituitary-adrenal (HPA) axis is an important component of the adaptive response to stress (Dayas et al., 2001; Day and Walker, 2007; Ulrich-Lai and Herman, 2009). In this regard it is noteworthy that the neuroendocrine paraventricular nucleus (PVN), the apex of the HPA axis, contains both OxR1 and OxR2 and that both receptor sub-types are expressed in the anterior and intermediate lobe of the pituitary gland (Trivedi et al., 1998; Date et al., 2000). Consistent with this anatomical evidence, several reports have suggested that orexins can modulate the HPA axis. For example, i.c.v. injections of orexin have been shown to increase Fos-protein expression in PVN corticotropin-releasing factor (CRF) neurons (Sakamoto et al., 2004), provoke adrenocorticotropin-releasing hormone (ACTH) release from the anterior pituitary, and increase the release of corticosterone from the adrenal glands (Jászberényi et al., 2000; Kuru et al., 2000; Russell et al., 2001; Moreno et al., 2005).

Importantly, researchers using systemic administration of orexin receptor antagonists have reported less consistent effects on HPA axis activity as would have been predicted from studies assessing HPA axis activity after orexin peptide infusions. For example, systemic injections of the OxR1 antagonist GSK-1059865 did not alter corticosterone responses to the pharmacological stressor yohimbine in rats (Gozzi et al., 2011, 2013). Further, oral treatment with almorexant had no effect on basal, social interaction, novelty or restraint stress-induced corticosterone release (Steiner et al., 2013b). Systemic SB-334867 administration also did not attenuate withdrawal-induced increases in plasma corticosterone release (Laorden et al., 2012) despite reducing the physical symptoms associated with morphine withdrawal in Wistar rats.

These equivocal effects of orexin on HPA axis function are somewhat surprising given the abundance of orexin receptors in the PVN and pituitary (Trivedi et al., 1998). Interestingly, i.c.v. administration of TCS-OX2-29, a selective OxR2 antagonist, attenuated swim stress-induced increases in plasma ACTH release (Chang et al., 2007) and, intra-PVT infusions of SB-334867 attenuated the ACTH response to restraint but only following repeated swim stress (Heydendael et al., 2011). Thus, any role of orexin in HPA axis control might depend on prior stress exposure, the category of stressor e.g., physical vs. psychological, its intensity and duration, or whether in repeated stress experiments, homotypic or heterotypic stressors are applied.

Despite the above data, exposure to psychological and physical stressors increase surrogate indices of orexin system function. For example, increased Fos-protein expression is observed in orexin neurons following exposure to acute footshock (Harris and Aston-Jones, 2006), fear-associated contexts and novel environments (Furlong et al., 2009). Interestingly, similar effects are not observed following acute immobilization stress (Furlong et al., 2009), however this form of stress does increase orexin mRNA levels in the LH (Ida et al., 2000). An explanation for these contrasting findings remains to be determined, however, one interpretation is that only sufficiently intense or salient stimuli recruit the orexin system and that prior arousal state strongly influences the likelihood of orexin system recruitment. With respect to pharmacological stressors, increased Fos-expression is observed in orexin neurons following systemic injections of the anxiogenic drug FG-7142 (Johnson et al., 2012), caffeine (Johnson et al., 2012) and intravenous administration of sodium lactate (Johnson et al., 2010). Additionally, systemic administration of OxR1 antagonist GSK-1059865 has demonstrated functional inhibition in stress-relevant brain regions such as the NAC, dorsal thalamus, amygdala, and ventral hippocampus following the administration of yohimbine (Gozzi et al., 2013).

Acute stress also appears to have long lasting effects on orexin gene expression. For example, 2 weeks following a single session of footshock stress, increased prepro-orexin mRNA levels were observed in both the medial and lateral divisions of the hypothalamus (Chen et al., 2013). Elevated levels of orexin-A peptide in the cerebrospinal fluid (CSF) of Wistar rats have also been demonstrated following a short-term forced swimming paradigm (Martins et al., 2004). In addition, increased orexin mRNA was observed in rats immediately following morphine withdrawal (Zhou et al., 2006). Consistent with this Fos-activity mapping of orexin cell reactivity to stress, acute orexin peptide infusions evoke anxiety-like behavior. Specifically, i.c.v. administration of orexin-A produced anxiogenic-like effects i.e., increased the time spent in the closed arms of the elevated plus maze and the time spent in the dark compartment of the light-dark test (Suzuki et al., 2005). Orexins have also been shown to modulate the activity of extrahypothalamic CRF systems with i.c.v. injections of orexin-A found to increase the percentage of CRF cells that express Fos in the central amygdaloid nucleus (Sakamoto et al., 2004). Together, this evidence forms the basis for the hypothesis that manipulation of the orexin system using SORAs and DORAs will likely also impact on stress–induced anxiety-like behavior.

## Effect of SORAs and DORAs on acute behavioral stress reactivity and anxiety-like behavior

Several recent preclinical studies indicate that SORAs and DORAs have a limited effect on basal/non-stress evoked behavioral responses including anxiety-like behavior. For example, systemic treatment with SB-334867 in both rats and mice had no effect on activity in the elevated plus maze and social interaction task, two common tests of anxiety-like behavior (Johnson et al., 2010; Rodgers et al., 2013). However, intra-PVT SB-334867 infusions resulted in decreased anxiety-like behavior in the elevated plus maze (Heydendael et al., 2011). This could indicate an important role for orexin signaling in the PVT in regulating basal arousal or anxiety state.

Importantly, SORAs reliably attenuated anxiety-like behavior evoked by an acute psychological or physical stressor. For example, systemic SB-334867 administration reduced the expression of anxiety-like behavior induced by acute nicotine (Plaza-Zabala et al., 2010) and sodium lactate injections (Johnson et al., 2010) as well as cat odor (Staples and Cornish, 2014). Administration of SORAs also reduced physiological responses such as increased heart rate and body temperature produced by exposure to acute i.p injection stress (Rusyniak et al., 2012). With regards to OxR2 antagonists, intra-PVT infusions of TCS-OX2-29 reduce anxiety-like behavior elicited by footshock stress (Li et al., 2010) suggesting a role for orexin signaling through the OxR2 in stress-relevant behaviors.

There are also several recent animal studies that have explored the role for the orexin system in anxiety-like behavioral responses to conditioned cues that predict the presentation of a fearful stimulus, such as footshock. This approach has been used to explore brain mechanisms responsible for generating panic disorder and post-traumatic stress disorder in humans (Grillon, 2008). In male Sprague-Dawley rats, Sears et al. (2013) administered SB-334867 i.c.v. before fear conditioning and demonstrated an impairment in conditioned stimulus (CS)-induced freezing behavior 24 h after CS/unconditioned stimulus (US) presentation. Further, systemic injections of TCS-1102 decreased conditioned and generalized fear and anxiety-like behavior in an open field in rats previously exposed to footshock stress (Chen et al., 2013). Moreover, OxR1 knockout mice demonstrated a reduction in freezing behavior in both cued and contextual fear conditioning. Interestingly, OxR2 knockout mice also showed reduced freezing behavior to contextual but not conditioned fear (Soya et al., 2013). Consistent with a role for both receptor subtypes in aspects of conditioned fear responses, Steiner et al. (2012) reported that oral administration of almorexant reduced fear-potentiated startle in response to a CS but found no change in elevated plus maze behavior. Interestingly, human genetic studies have linked a polymorphism in Orx-2 receptor gene with panic disorder (Annerbrink et al., 2011).

Together these data indicate that exposure to acute stress and the expression of anxiety-like behavior is generally associated with increases in orexin system activity. Further, orexin antagonists appear to be potential candidates for the suppression of anxiety-like behavior and maladaptive stress-reactivity. Importantly, as is outlined below, recent evidence suggests that chronic suppression of the orexin system may be associated with the development of depression-like symptomology. These findings have significant implications for the potential use of orexin antagonists in the treatment of neuropsychiatric disorders.

## Effect of chronic stress on the orexin system and implications for depression

In recent human studies, a polymorphism in Orx-1 receptor gene has been associated with major mood disorders and increased orexin peptide levels were correlated with positive emotions and social interaction (Rainero et al., 2011; Blouin et al., 2013). Non-genetic factors, such as chronic stress, have also implicated orexin in the etiology of depression (Katz et al., 1981; Kendler et al., 1999; Charney and Manji, 2004; Russo and Nestler, 2013). This link has been studied in animal models typically involving extended periods of psychological stress exposure (Lutter et al., 2008). Increasing evidence from these types of studies links chronic stress exposure to a downregulation of orexin system activity. For example, mice exposed to the well characterized social defeat model of chronic stress, which evokes symptoms thought to mimic depression in humans, display reduced orexin mRNA expression, lowered orexin cell number, and diminished levels of orexin A and orexin B peptide (Lutter et al., 2008; Nocjar et al., 2012). Similarly, Wistar-Kyoto (WKY) rats, which exhibit a depressive-like behavioral phenotype, express lower numbers of orexin neurons and a smaller orexin soma size compared to Wistar rats (Allard et al., 2004). The WKY rat strain also exhibits reduced prepro-orexin mRNA levels and orexin A immunoreactivity is reduced in the hypothalamus, thalamus, septum and amygdala (Taheri et al., 2001).

Early life stress (ELS) is a known risk factor for the development of stress-related mood disorders in adulthood (Graham et al., 1999). Surprisingly though, work has shown that ELS in fact increases frontal cortical OxR1 and hypothalamic orexin A levels in adulthood (Feng et al., 2007). Given these results, we recently investigated the effects of ELS on the reactivity of the orexin system to a second psychological stressor in adulthood. Both male and female rats exposed to neonatal maternal separation displayed a hypo-active orexin cell response to stress in adulthood. These animals also displayed reduced open field behavior but notably no overt anxiety-like behavior was observed on an elevated plus maze. Our interpretation of these data is that the ELS procedures evoked behaviors more akin to a depression-like profile after stress however further behavioral tests will be necessary to further explore this hypothesis (Campbell et al., 2013).

What effect a second stressor, such as restraint in adulthood, might have on LH-orexin circuits is unknown. This would seem important given the behavioral effects of this “two-hit” paradigm outlined above (Campbell et al., 2013). It is possible that increased drive to this system reflects an attempt to enhance orexin activity and prevent the expression of depression-like behavior. It may be that in response to a significant subsequent stressor, the reduced functional integrity at a synaptic and molecular level is unmasked, which may then develop into a depressive-like state. Regardless, taken together with the Fos data outlined above, our findings suggest that ELS can have long-term impacts on the normal functioning of orexin cells.

Consistent with a process where ELS can induce orexin cell dysfunction and depression-like behaviors, clinical evidence supports the possibility that decreased orexin signaling might promote depression-like behaviors. For example, reduced concentrations of orexin A were reported from CSF samples of adults with major depressive disorder and chronic, combat-related post-traumatic stress disorder (Brundin et al., 2007a,b, 2009; Strawn et al., 2010). Additionally, reduced orexin A mRNA has been correlated with increased scores on the Hamilton rating scale for depression (Rotter et al., 2011). Interestingly, infusions of orexin peptides into the ventricles of rats have been shown to have antidepressant-like effects. For example, i.c.v. administration of orexin reduces the duration of immobility in the forced swim test, and this effect is blocked by the administration of the OxR1 antagonist, SB-334867 (Ito et al., 2008). Thus, it is possible that in some forms of depression, increasing orexin system signaling may have therapeutic benefit. In line with this interpretation, we recently found that a period of exercise in adolescence prevented reductions in orexin system function and the expression of stress-related behavior in rats exposed to ELS (Campbell et al., 2013).

Despite the above work, it is important to acknowledge that there are both preclinical animal and human data that do not support a link between a hypoactive orexin system and depression. For example, Mikrouli et al. (2011) demonstrated that the Flinders Sensitive Line, considered a genetic rat model of depression, displayed elevated, not depressed, levels of orexin neurons compared to controls. Further, Nollet et al. (2011) demonstrated that mice subjected to unpredictable chronic mild stress displayed increased depressive-like behavior following the tail suspension test but presented with elevated orexin neuron activity in the DMH and PFA subregions of the hypothalamus. Interestingly, these authors were able to reverse this elevation in orexin cell activity with 6 weeks of fluoxetine treatment. Similarly, exposure to unpredictable chronic mild stress produced depressive-like behaviors in the tail suspension test, elevated plus maze and resident-intruder task; and 7 weeks exposure to the DORA almorexant produced an antidepressant-like behavioral effect in these tasks (Nollet et al., 2012). And, in a recent study, OxR1 mRNA expression in the amygdala was reported to be positively correlated with increased depressive-like behavior in the forced swim test (Arendt et al., 2013) - however it is possible that this effect might be caused by downregulated orexin system function. Finally, a recent study reported that decreased depressive-like behavior is observed in OxR1 knockout mice, whereas OxR2 knockout mice exhibit *increased* depressive-related behavior, possibly pointing to a differential role for OxR1 vs. OxR2 in the regulation of these behaviors (Scott et al., 2011). These authors highlight the fact that behavioral pharmacology studies typically use the non-selective OxR agonist orexin-A, along with SORAs at doses that are potentially non-selective *in vivo*, therefore making it difficult to differentiate roles for OxR1 vs. OxR2 in the regulation of depression-like behavior.

In human studies, Salomon et al. (2003) reported that orexin A CSF levels were higher in depressed patients and treatment with sertraline, an antidepressant drug, resulted in an attenuation of CSF orexin levels. Furthermore, a positive correlation between orexin plasma concentrations, depressive symptoms and global distress indices on the brief symptom inventory is seen following alcohol withdrawal (von der Goltz et al., 2011). Finally, Schmidt et al. (2011) failed to find any association between CSF orexin A levels and depression.

Taken together, these results suggest that acute stress may activate the orexin system in order to enhance an animal's ability to cope or adapt appropriately to a potential threat (Figure 1C). If these stressors persist, chronic or repeated exposure to stress may downregulate orexin system function (Figure 1D). Hypoactivity of the orexin system may impair an animal's ability to adapt to stress and lead to the expression of depressive-like behavior. Data from human and animal studies not supporting this link may reflect the heterogeneous nature of depression—i.e., depression presenting with and without anxiety or anxiety presenting with or without depression. Supporting this conclusion, Johnson et al. (2010) found that patients exhibiting panic anxiety displayed increased CSF orexin levels compared to patients exhibiting panic anxiety with comorbid major depressive disorder. It will be important for human and animal studies exploring the link between the orexin system dysfunction and neuropsychiatric conditions to consider the heterogeneous nature of these conditions.

## Potential pitfalls for approaches to treat neuropsychiatric disorders using orexin receptor antagonists

As outlined above, significant progress has been made in our understanding of the contribution of the orexin system to normal and “pathological” behavior. In the case of addiction, there seems sufficient evidence to conclude that the orexin system is important for drug-seeking behavior, particularly for relapse-like behavior provoked by drug-cues and stress but not by drug itself. Further, several studies indicate that orexin antagonists, and in particular selective OxR1 antagonists, can reduce drug-seeking at doses that have minimal effects on natural reward-seeking behavior (Jupp et al., 2011; Hollander et al., 2012; James et al., 2012; Brown et al., 2013). Comparison of orexin's role in drug taking vs. seeking behavior also highlights that orexin receptor antagonists have effects on rewarded self-administration, but importantly these effects generally emerge only under high effort schedules of reinforcement. These data combine to produce a compelling case that orexin receptors represent promising targets for the treatment of addiction. This is particularly true given the long-established interaction between addiction and maladaptation of natural reward seeking brain pathways. For example, increased excitatory drive to the orexin system is thought to heighten orexin signaling in key reward-seeking regions such as the VTA and may contribute to the persistent plasticity within dopamine neurons seen after long-term cocaine self-administration and increased relapse vulnerability (Chen et al., 2008; James et al., 2012)

It is important given the clinical promise of SORAs and DORAs for neuropsychiatric disorders that studies continue to assess the potential for off-target effects, tolerance to prolonged orexin receptor blockade and differential or counter-regulatory effects on OxR1 vs. 2, as well as any possible compensatory adaptations that may occur in other hypothalamic neuropeptide systems in response to chronic orexin receptor blockade. Studies to date indicate a limited profile of chronic SORA and DORA tolerance. For example, Steiner et al. (2013c) showed that 12 days of chronic almorexant treatment had no effect on the maintenance of CPP or locomotor sensitization in animals exposed to cocaine, morphine or amphetamine. However, as mentioned above, Nollet et al. (2012) exposed mice to chronic almorexant treatment (7 weeks), which produced an antidepressant-like effect in the tail suspension test, elevated plus maze and resident-intruder task following exposure to unpredictable chronic mild stress. Interestingly, chronic treatment with the OxR1 antagonist ACT-335827 (4 weeks) did not alter total energy intake in cafeteria diet fed rats compared to controls (Steiner et al., 2013b). In contrast, chronic SB-334867 treatment (14 days) had anti-obesity effects in a model of genetically obese mice by reducing food intake and body weight gain over the 14 day period (Haynes et al., 2002).

In one of the few examples where repeated doses of an orexin antagonist have been studied for addiction and relapse prevention, Zhou and colleagues demonstrated that chronic SB-334867 exposure resulted in a complex pattern of effects (Zhou et al., 2011). Specifically, they found that repeated SB-334867 exposure prior to extinction sessions resulted in reduced cocaine-seeking behavior in rats during extinction; however, repeated SB-334867 treatment during extinction increased cue-induced reinstatement and had no effect on cocaine-primed reinstatement. Importantly, McNally and colleagues have also shown that cocaine and alcohol-seeking do not necessarily evoke a specific drug context-related activation of the orexin system, rather, recruitment of this pathway is necessary but not sufficient for drug-seeking behavior (Hamlin et al., 2007, 2008). It is also interesting to note that the OxR1 antagonist, SB-334867, has been shown to reduce cue-induced reinstatement of cocaine seeking behavior in male rodents yet no effect was seen in female rats (Zhou et al., 2012). Future work should focus on differentiating arousal vs. reward related function of the orexin neurons in the context of addiction. Sex-specific effects of stress and drug exposure on orexin circuitry also warrant further scrutiny.

With respect to the potential use of orexin receptor antagonists for anxiety and depression, recently, Johnson et al. (2010, 2012) proposed the use of OxR1 antagonists in the treatment of panic disorder. Certainly, the available data appears to support an important role for orexin signaling in ameliorating anxiety-like states in animal models. Studies employing chronic stress paradigms, however, suggests that a more complicated picture exists and that the effects of repeated SORA or DORA treatment may be unpredictable (Zhou et al., 2012). Emerging data indicates that ELS and chronic stress can downregulate the activity of the orexin system in response to chronic stress (Lutter et al., 2008; Nocjar et al., 2012; Campbell et al., 2013). There is also a developing clinical literature indicating that depression may be associated with decreased orexin system function (Brundin et al., 2007a,b, 2009). These studies raise potential concerns for the long-term use of orexin antagonists in the treatment of addiction and anxiety disorders, as long-term suppression of the orexin system may precipitate depressive-like symptoms. Similarly, the emergence of anxiety or depression under conditions of augmented orexin system function will need to be carefully considered. Therefore, a greater understanding of the changes to orexin receptor expression in relevant brain regions will be necessary to confidently predict the potential outcomes of therapeutic manipulation of orexin signaling under these conditions. Further we propose that a thorough evaluation of chronic and subchronic orexin receptor antagonism in preclinical animal models of anxiety and depression is necessary. Together, such approaches may be able to identify therapeutic dosing regimens with preferential effects on the different aspects for the orexin system that influence mood, as well as drug- and natural-reward behaviors. Finally, the evidence that drugs of abuse or stress can rewire inputs onto orexin neurons indicates that targeting these changes within the LH might offer an alternative strategy. This would negate the mixed downstream effects of SORAs and DORAs in mood, addiction and reward by reducing aberrant orexin cell activity at the site of dysfunction, rather than simply masking the downstream effects using antagonists. For example, our group is currently investigating the changes in LH-orexin circuits responsible for the increased excitatory drive observed after cocaine or ELS.

In conclusion, significant progress has been made toward an understanding of the role of the orexin system in normal and pathological behaviors. Unsurprisingly, given the widespread projections of the orexin system, the role for these neurons crosses many domains including basic physiological responses to more complex functions. Our view is that only a comprehensive dissection of the changes in LH-circuit function, both within the hypothalamus and in target regions of these neurons, will reveal appropriate therapeutic avenues to augment or suppress dysregulated orexin function in neuropsychiatric and neurological disease states.

### Conflict of interest statement

The authors declare that the research was conducted in the absence of any commercial or financial relationships that could be construed as a potential conflict of interest.
